# Many-qubit protection-operation dilemma from the perspective of many-body localization

**DOI:** 10.1038/s41467-022-33657-3

**Published:** 2022-10-03

**Authors:** Matti Silveri, Tuure Orell

**Affiliations:** Nano and Molecular Systems Research Unit, University of Oulu, Oulu, Finland

**Keywords:** Quantum information, Phase transitions and critical phenomena, Qubits

## Abstract

What is an optimal parameter landscape and geometric layout for a quantum processor so that its qubits are sufficiently protected for idling and simultaneously responsive enough for fast entangling gates? Quantum engineers pondering the dilemma might want to take a look on tools developed for many-body localization.

Quantum computing algorithms can be seen as a temporally and spatially scattered collection of high-fidelity quantum gates operated on a large array of qubits. The first requirement for successful computing is fast single-qubit gates with high precision. Here, we encounter the single-qubit protection-operation dilemma: How to both protect the qubit against environmental errors and simultaneously operate it efficiently. In other words, it would be ideal to isolate a qubit so that no disturbance would harm the delicate quantum state within it. But when operating the qubit, the protection needs to be at least partially lifted for the external controlling pulses to enter. This dilemma has been one of the key focus points of the development of superconducting qubits since the early days. After over 20 years of progress, the single-qubit gate fidelities have been pushed to the level of 99.99%^1^.

## Many-qubit protection-operation dilemma

In quantum processors made of tens or hundreds of qubits, we face another—and possible even trickier—version of the protection-operation dilemma. It is rooted in qubit-qubit interactions and fundamentally related to the propagation of quantum information. In the single-qubit dilemma, the environment is an external one, formed, by electromagnetic radiation, fluctuations in materials, or other disturbances. In the many-qubit dilemma, the environment is an internal one, formed by all the other coupled qubits in the quantum processor. A solution to completely bypass the dilemma is to engineer tunable qubit-qubit couplings such that interaction with the problematic internal environment can be turned on and off on demand. Unfortunately, tunable couplers increase hardware complexity, and thus might be an unfavorable architectural direction as the number of qubits scales up. We focus here on non-tunable settings to highlight the profound design challenges of quantum processors.

**Fig. 1:**
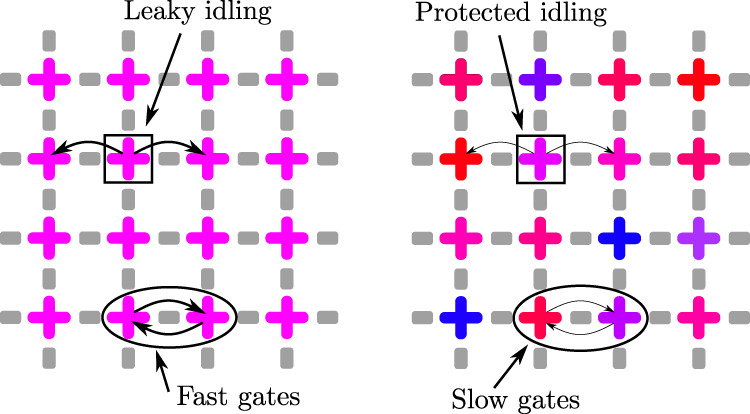
Many-qubit protection-operation dilemma. With no frequency disorder in qubits, one can apply fast two-qubit gates but the idle qubits rapidly leak quantum information. On the other hand, in sufficiently disordered systems, the quantum states are quasi-localized, rendering the idle qubits well protected. A downside is that the two-qubit gates are less efficient. Crosses visualize superconducting qubits and their colors indicates transition frequencies. Gray bars denote qubit-qubit interactions.

The second requirement for successful quantum computing is the ability to perform high-fidelity entangling operations between qubits, such as CNOT gates. Creating entanglement is typically realized by combining a physical qubit-qubit interaction, such as direct capacitive coupling, with driving control pulses^1^. To minimize the overall effect of dissipation and decoherence we would like to have the fastest possible gates. Qubits that are identical in their transition frequency entangle with the rate that is set by their coupling strength. If the qubit frequencies are non-identical, the time for creating full entanglement between them increases quickly, essentially proportional to their frequency detuning. In short, for operating a qubit array, it would be ideal to have identical and strongly coupled qubits, see Fig. 1.

Not all qubits are subject to active gates all the time. That is why the third requirement for successful quantum algorithms is high-fidelity idling. Actually, idling is also a quantum gate, known as the identity operation. Somewhat non-intuitively, realizing the identity gate in high-fidelity is quite a non-trivial task. Subjecting the idling qubits to dynamical decoupling pulses^2^ or active quantum error correction methods^3^ can provide substantial fidelity improvements assuming that the used gate and control pulses are good enough. Our focus is on the static point of view instead.

An ideal setting for idling would be such where qubits have negligible or very weak effective mutual interactions. In this case, quantum information travels poorly between the qubits and gate operations between nearby qubits have no essential effect on the idling qubits. When the qubits have fixed physical coupling mechanisms then one can achieve weak effective interaction by strongly detuning the qubit frequencies, see Fig. 1. In an array with many qubits, all of them need to be detuned with each other, creating a disorder landscape.

What is an optimal amount of frequency disorder so that a qubit array is sufficiently protected in terms of idling and simultaneously responsive enough for fast high-fidelity entangling gates? Does geometry or dimensionality play a role? Layouts of the state-of-the-art quantum processors^4–6^ vary considerably in geometry, overall coupling strengths, and the magnitude of frequency disorder, suggesting that the many-qubit protection-operation dilemma is yet to be solved.

## Sharp measures for the tension between optimally protecting and efficiently operating

The recent work^7^ by Berke et al. offers sharp tools to gauge the tension between optimally protecting and efficiently operating a quantum processor. Their physical intuition and insights are based on the physics of many-body localization^8^ studying the interplay of disorder and interactions in non-equilibrium many-body quantum systems.

If a conventional generic many-body system experiences a local perturbation, then information on this event spreads rapidly within the whole system. Eventually the system settles into a smooth thermal equilibrium according to principles of statistical physics. In other words, conventional systems belong to a chaotic phase where the many-body eigenstates extend throughout the system volume and quench dynamics lead to a rapid spreading of entanglement. However, with sufficiently strong disorder, the system leaves the chaotic phase and undergoes a transition to the many-body localized phase. Then the eigenstates are quasi-localized, and information as well as entanglement propagate only logarithmically slowly beyond local regions.

The main invention of Berke et al. is to relate the concepts of many-body localization to the many-qubit protection-operation dilemma. Quantum processors should be designed to be sufficiently disordered so that they are in the localized phase in order for the effect of local quantum gates to stay local. Too much disorder is not advantageous since it means going too deep in the localized phase and slowing two-qubit entangling gates unnecessarily. Too little disorder means going too close to the chaotic phase, leading to fluctuations in idling qubits by gates and dynamics in other qubits.

**Fig. 2:**
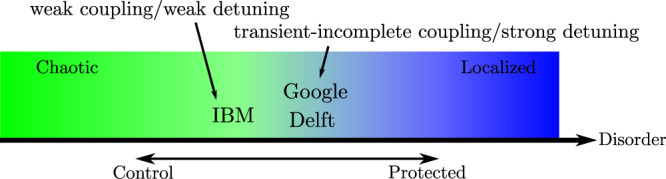
A schematic of the tension map between optimally protecting and efficiently operating. Utilizing the measures based on the many-body localization, we see that different quantum processor manufacturers have settled upon varying architectural design protocols with both benefits and downfalls with respect to control and protection. The schematic is based on results of ref. 7.

The literature of many-body localization^8,9^ knows several sharp measures to distinguish the phase transition from the chaotic to the localized phase. By utilizing openly available data on design parameters of superconducting quantum processors, Berke et al. constructed the Hamiltonians and numerically computed the eigenenergy and eigenstate spectra for the devices. Their first measure is to compare the statistics of the energy levels in the quantum devices to the known statistics in the many-body localized phase (Poisson statistics) and in the chaotic phase (Wigner-Dyson statistics) via Kullback–Leibler divergence. The second measure is the inverse participation ratio gauging the variance of the wavefunctions in the basis of local qubit occupations (Fock basis). If a system is localized then the many-body wavefunctions involve only a few Fock basis coefficients and if it is chaotic then the wavefunctions are generically superpositions of many Fock basis states. Based on these measures, different quantum processors designs are put on a tension map between optimally protecting and efficiently operating, see Fig. 2. One of their main results is that the current processor designs are pretty close to the dangerous chaotic phase, and it might not be good a idea to reduce qubit disorder any further.

Furthermore, Berke et al. invented a new, even sharper, diagnostic tool based on the statistics of the actual residual (ZZ) couplings. It relies on the idea of first considering an eigensystem of non-coupled qubit array, then gradually turning on the physical qubit-qubit couplings and tracking how the non-coupled energy levels are transformed into the many-body energy levels of the so-called localized qubits. If the disordered and interacting system stays in the many-body localized phase, then the effective residual (ZZ) interaction strength between the localized qubits decreases exponentially as a function of the physical distance of the localized qubits^8,9^. From the point of view of quantum processor design, exponential decay in residual couplings means efficient protection. Berke et al. developed an effective numerical method for both tracking the evolution of the eigensystem with increased couplings and showed that the residual coupling coefficients can be straightforwardly identified via the Walsh-Hadamard transformation applied on the energy levels.

## Quantum processors as simulators of disordered quantum matter

We can also look at the topic from the opposite point of view. What can large qubit arrays teach us on the physics of many-body localization? Manufacturing identical superconducting circuits is challenging. As these devices naturally contain some disorder, why not to study the physics of disordered quantum matter with them. Furthermore, the anharmonicity, which allows one to use superconducting circuits as qubits in the first place, can be interpreted as a many-body interaction, which is essential for the formation of the many-body localized phase. Thus, superconducting qubit arrays provide a natural platform for the experimental study of the intricacies of the many-body localization^10^. Exact numerical modeling of disordered quantum matter is limited to roughly 20 qubits, raising the question of what is the possible role of finite-size effects. Nowadays, much larger experimental systems are available^4,5^, and thus a transition from numerical simulations to experimental quantum simulations can shed more light on the subject. The topic has already been experimentally studied in systems with nine to nineteen^11–13^ transmon qubits in good unison with the available theoretical predictions.

## Outlook

The work by Berke et al. is an exemplar on a beneficial and constructive symbiosis between a fundamental theoretical research field and a more applied research field, in this case between quantum many-body physics and the engineering of superconducting quantum processors. The essential concepts here are the structure, dynamics, and control of quantum states and entanglement. It will be fascinating to see which kind of applications other modern topics of quantum many-body dynamics, such as measurement-induced phase transitions^14^ or dynamical quantum phase transitions^15^ might find in quantum device engineering or in other fields where controlling quantum entanglement is essential for applications.
